# Cholesterol metabolism in aging simultaneously altered in liver and nervous system

**DOI:** 10.18632/aging.203880

**Published:** 2022-02-07

**Authors:** Valéria Sutti Nunes, Guilherme da Silva Ferreira, Eder Carlos Rocha Quintão

**Affiliations:** 1 Laboratorio de Lipides (LIM10), Hospital das Clinicas HCFMUSP, Faculdade de Medicina, Universidade de Sao Paulo, Sao Paulo, Bazil

**Keywords:** cholesterol, 24-hydroxycholesterol, aging, liver, brain

## Abstract

In humans, aging, triggers increased plasma concentrations of triglycerides, cholesterol, low-density lipoproteins and lower capacity of high-density lipoproteins to remove cellular cholesterol. Studies in rodents showed that aging led to cholesterol accumulation in the liver and decrease in the brain with reduced cholesterol synthesis and increased levels of cholesterol 24-hydroxylase, an enzyme responsible for removing cholesterol from the brain. Liver diseases are also related to brain aging, inducing changes in cholesterol metabolism in the brain and liver of rats. It has been suggested that late onset Alzheimer’s disease is associated with metabolic syndrome. Non-alcoholic fatty liver is associated with lower total brain volume in the Framingham Heart Study offspring cohort study. Furthermore, disorders of cholesterol homeostasis in the adult brain are associated with neurological diseases such as Niemann-Pick, Alzheimer, Parkinson, Huntington and epilepsy. Apolipoprotein E (apoE) is important in transporting cholesterol from astrocytes to neurons in the etiology of sporadic Alzheimer’s disease, an aging-related dementia. Desmosterol and 24S-hydroxycholesterol are reduced in ApoE KO hypercholesterolemic mice. ApoE KO mice have synaptic loss, cognitive dysfunction, and elevated plasma lipid levels that can affect brain function. In contrast to cholesterol itself, there is a continuous uptake of 27- hydroxycholesterol in the brain as it crosses the blood-brain barrier and this flow can be an important link between intra- and extracerebral cholesterol homeostasis. Not surprisingly, changes in cholesterol metabolism occur simultaneously in the liver and nervous tissues and may be considered possible biomarkers of the liver and nervous system aging.

## INTRODUCTION

Over the last few decades, there has been an increase in the elderly population. Between 2015 and 2050 the number of people aged 60 and over is expected to double worldwide (https://www.who.int/news-room/fact-sheets/detail/ageing-and-health). The aging process is accompanied by numerous pathological changes and understanding them can help us improve the available treatments aimed at improving the quality of life.

Specifically, in lipid metabolism, advancing age is associated with a gradual increase in plasma concentrations of triglycerides (TG), cholesterol and low-density lipoprotein (LDL) [1]. It is suggested that the reduction in the concentration of total cholesterol in some studies with long-lived elderly individuals is due to the death of those with the highest blood cholesterol [2, 3]. These changes may contribute to the increased risk of cardiovascular, neurological and liver disease observed in the elderly.

Cholesterol is one of the main components of the cell plasma membrane, giving it its physicochemical properties, such as fluidity and stability, however cholesterol is not evenly distributed in the membranes. It focuses on specialized sphingolipid-rich domains called lipid rafts and caveolae, which are involved in important cellular functions such as signaling across membranes [4, 5], regulation of membrane traffic, and signal transduction pathways that initiate in the membrane by stimulation or dimerization of receptors [6].

In addition to structural functions, enzymatic and non-enzymatic liver pathways give rise to several oxysterols, some of which are further metabolized into bile acids [7]. Oxidative cleavage of the cholesterol side chain generates pregnenolone, the common precursor to all other steroid hormones [8].

The body’s cholesterol content is influenced by its ingestion, absorption, synthesis and excretion. Coordinately, modifications in one of these components lead to responses from the others in order to maintain cholesterol homeostasis. For example, the approximately four-fold increase in dietary intake of cholesterol reduced its synthesis, assessed in plasma lymphocytes, by 34% [9].

**Figure 1:**
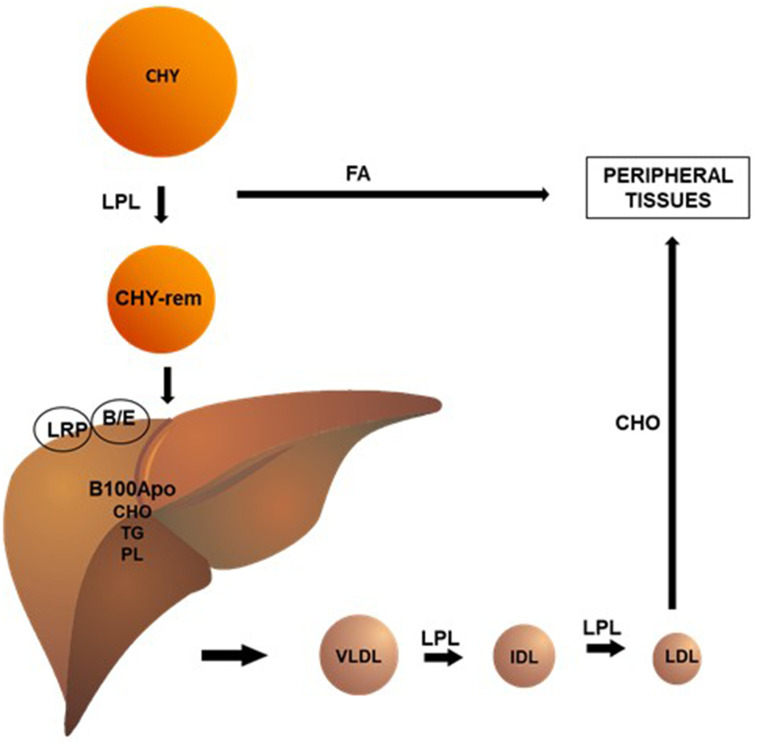
**Chylomicrons (CHY) undergo the action of the lipoprotein lipase (LPL) enzyme, which hydrolyzes triglycerides (TG) and phospholipids (PL) present in these lipoproteins, making them available to peripheral tissues.** This process reduces the size of the CHY, forming remnant CHY, which are quickly taken up by the B/E receptors and proteins related to the hepatic LDL receptor (LRP). The liver, in turn, synthesizes very low-density lipoprotein (VLDL) by conjugating TG (triglycerides), PL (phospholipids), and CHO (cholesterol) to apolipoprotein (apo) B100. Analogously to CHY, VLDL undergo LPL action in the circulation, originating intermediate density lipoproteins (IDL) and, ultimately, low-density lipoproteins (LDL). In human blood, LDL is the main carriers of cholesterol to peripheral tissues.

In blood vessels, chylomicrons (CHY) undergo the action of the lipoprotein lipase (LPL) enzyme, which hydrolyzes triglycerides (TG) and phospholipids (PL) present in these lipoproteins, making them available to peripheral tissues. This process reduces the size of the CHY, forming remnant CHY, which are quickly taken up by the B-E receptors and proteins related to the hepatic LDL receptor (LRP). The liver, in turn, synthesizes very low-density lipoprotein (VLDL) by conjugating TG, PL and cholesterol to apolipoprotein (apo) B100. Analogously to CHY, VLDL undergo LPL action in the circulation, originating intermediate density lipoproteins (IDL) and, ultimately, low-density lipoproteins (LDL). In human blood, LDL is the main carriers of cholesterol to peripheral tissues (Figure 1).

**Figure 2:**
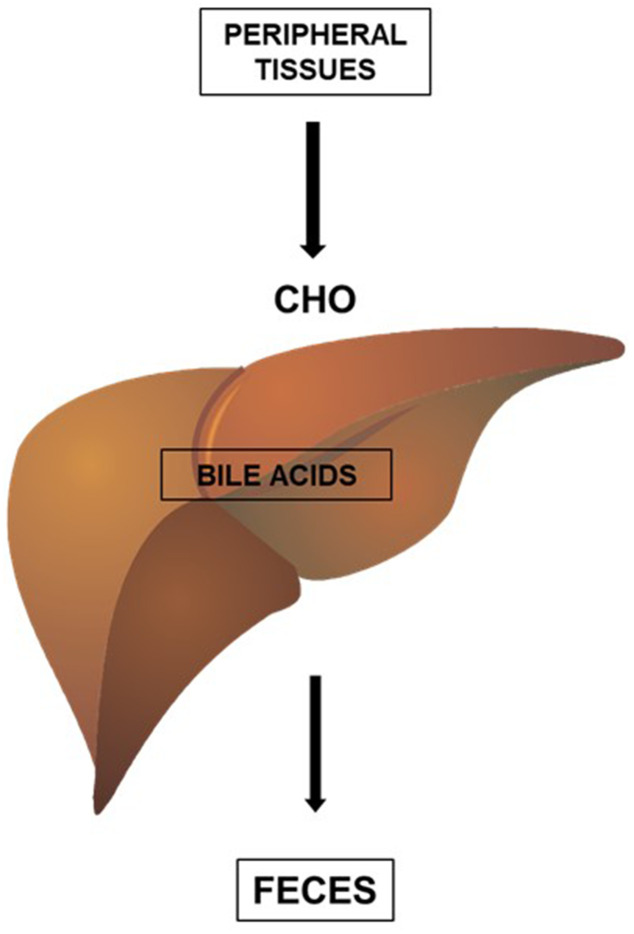
**HDL promotes the reverse transport of cholesterol (CHO), a process by which cholesterol (CHO) is removed from peripheral tissues and delivered to the liver for elimination in the feces.** The excretion of cholesterol by the liver, in the form of bile acids, is an important form of elimination of cholesterol from the body.

High-density lipoproteins (HDL) can be formed during the lipidation of apoA1, a protein synthesized in the liver and intestine, or during the metabolism of CHY and VLDL [10]. The main function of HDL is to promote the reverse transport of cholesterol, a process by which cholesterol is removed from peripheral tissues and sent to the liver for elimination in the feces. The excretion of cholesterol by the liver, in the form of bile acids, is the main form of elimination of cholesterol from the body (Figure 2).

The liver is the main organ responsible for synthesizing and eliminating excess cholesterol from the body. Cholesterol synthesis involves several molecular reactions and an efficient feedback control mechanism. The main regulatory step in cholesterol biosynthesis is the reduction of 3-hydroxy-3-methylglutaryl coenzyme A (HMG-CoA) to mevalonate, a reaction catalyzed by HMG-CoA reductase, an enzyme incorporated in the membrane of the endoplasmic reticulum (ER). The activity of HMG-CoAR is regulated by different mechanisms. Most important is the control of the rate of synthesis of HMGCoAR mRNA by the family of sterol regulatory element binding proteins (SREBPs). SREBP-2 is abundant in the brain and liver, while SREBP-1 is restricted to the liver. Specifically, SREBPs induce transcription of the HMG-CoAR gene binding to the sterol regulatory element (SRE) in the DNA. When the cholesterol concentration is sufficient to maintain cell homeostasis, the SREBPs bind to the SREBP cleavage activating protein (SCAP) in the ER membrane. When the cellular cholesterol content decreases, the sterol leaves its binding site to SCAP and the SREBP-SCAP complex moves to the Golgi, where two proteases (S1P and S2P) release the amino terminal domain of the transcription factor. This amino terminal peptide enters the nucleus and binds the SREs. When the cytoplasmic concentration of cholesterol increases, the sterol molecules bind to SCAP and prevent the complex’s translocation to the Golgi, leading to a reduction in HMGCoAR transcription [8, 11].

Disturbances in cholesterol metabolism have been reported in several pathologies such as aging, diabetes mellitus, Alzheimer’s disease, multiple sclerosis, osteoporosis, lung cancer, breast cancer, and infertility [12, 13]. Furthermore, cholesterol metabolites may be more relevant than cholesterol itself in these pathologies [14–16].

## Changes in lipid metabolism with aging

The mechanisms of lipid metabolism alterations with aging are not fully understood. The loss of lean mass and the increase in adipose tissue stimulate insulin resistance and allow a greater flow of fatty acid to the liver, resulting in increased synthesis of VLDL [17] and diminished removal rate of these lipoproteins from the plasma [17–19]. This is due to impairments in the activity of lipoprotein lipase [20] and reduced concentration of the hepatic LDL receptor secondary to the increase in PCSK9 [21], a protein responsible for signaling LDLr degradation. The longer half-life of lipoproteins in plasma possibly exposes lipoproteins to greater chemical changes, which contribute to reducing their affinity for their receptor [22, 23].

Reverse cholesterol transport (RCT), carried out by HDL, is also diminished by aging. *In vitro*, it was observed that HDL isolated from elderly individuals had a lower capacity to remove cellular cholesterol when compared to HDL from 20-30 year old adults [24]. Recently, the *in vivo* RCT was evaluated in C57BL/6 mice at 3 or 20 weeks of age [25]. For this, J774 macrophages overloaded with labeled cholesterol were injected into the animals’ peritoneum. The recovery of cholesterol in plasma, liver and feces represented RCT. After 48 hours of macrophages inoculation, the authors observed that older animals had lower concentrations of labeled cholesterol in the three compartments studied, showing less efficiency in the process of cholesterol removal and excretion with aging. It is important to note that there was no difference between the groups according to the HDLc concentrations. The two studies mentioned above attributed the results to the damage caused by aging or by HDL in the elderly on transporters ABCA1 and ABCG1 receptors involved in the delivery of cholesterol to HDLs.

## Cellular export of cholesterol by the liver and nervous system

The liver is the only organ capable of metabolizing cholesterol into bile acids, representing approximately 1/3 of the cholesterol eliminated from the body. The enzymatic conversion of cholesterol into bile acids by the microsomal cytochrome P450 system is the most important mechanism for the removal of cholesterol from the body, while the generation of 24-hydroxycholesterol (24-HC) is the predominant form of cholesterol elimination by the central nervous system. The microsomal cytochrome P450 system is responsible for the enzymatic generation of oxysterols including 7α- hydroxycholesterol catalyzed by 7α-hydroxylase (CYP7A1) in the liver, 24-HC in the brain and retina catalyzed by 24S-hydroxylase (CYP46A1), and mostly 27-hydroxycholesterol (27-HC) of tissues catalyzed by 27-hydroxylase (CYP27A1) [26, 27].

As in the brain, cholesterol synthesis in the liver is strongly regulated by the activity of HMGCoAR, while peripheral cholesterol catabolism is mainly carried out by CYP27A1, via the production of 27-HC as the main catabolite. In contrast to cholesterol itself, there is a continuous uptake of 27-HC into brain since it crosses the blood–brain barrier [28]. While there is an efflux of 24-HC from the brain to the peripheral circulation, there is also an inflow of 27-HC to the brain [29]. The flux of 27-HC into the brain may be an important link between intra and extracerebral cholesterol homeostasis [28].

*In vitro* conditions and *in vivo* experiments with different mouse models showed negative effects of the 27-HC in brain that include memory defects [30], reduced production of the ‘memory protein’ Arc (activity-regulated cytoskeleton-associated protein) in hippocampus [31, 32] and reduced brain uptake of glucose [31].

## Cholesterol metabolism in the liver is altered in more frequent pathologies such as aging and non-alcoholic fatty liver disease

Other authors observed in human liver biopsies a negative correlation between age and 7α-hydroxycholestenone, a marker of bile acid synthesis, a result that was attributed to lower expression of the enzyme CYP7A1 [33]. Since less cholesterol is eliminated in the form of bile acids, bile cholesterol saturation increases with age, increasing the risk of gallstones. These results suggest a reduction in the export of the cholesterol content from the liver with aging. If, in humans, the low removal cell rate elevates hepatic cholesterol concentration, this could explain, at least in part, the reduction in hepatic LDL receptor concentration and plasma (and presumably also hepatic) cholesterol synthesis markers.

At least in murine models, hepatic cholesterol concentration increases as a function of age [34–36]. In animals, aging is accompanied by increased expression of Niemann-Pick C1-like 1 (NPC1L1) in the enterocyte [37], probably increasing the absorption of intestinal cholesterol eliciting increased liver cholesterol uptake [36]. However, other authors reported that in human livers age was associated with reduced bile acids synthesis attributed to decreased expression of nuclear factor-4 and, consequently, to low cholesterol 7alpha-hydroxylase activity [33].

The aging process predisposes to functional and structural hepatic impairment. The most common liver disease, which affects a third of the world’s population, is non-alcoholic fatty liver disease (NAFLD). This is characterized by the accumulation of fat in hepatocytes characterized as hepatic steatosis with active hepatic inflammation, known as non-alcoholic steatohepatitis. The prevalence of fatty liver disease has increased at alarming rates, along with obesity, diabetes and metabolic syndrome, becoming the second most common cause of cirrhosis after alcohol-related liver disease worldwide [38].

NAFLD patients present cholesterol metabolism alterations characterized by increased synthesis and decreased intestinal absorption of cholesterol [39] associated with liver fat content, regardless of body weight [39]. Alterations in cholesterol homeostasis pathways are linked to increased expression of HMGCR, and decreased expression of LDL receptors and bile acids synthesis [40].

NAFLD is a major health problem associated with obesity and metabolic syndrome, including insulin resistance and dyslipidemia. Lipid accumulation in hepatocytes causes liver damage and triggers inflammation, fibrosis and cirrhosis [41]. In addition to fatty acids and triglycerides, there is increased liver free cholesterol [42], with alterations in the cholesterol homeostasis pathways [40]. Measurements in mice livers indicate that cholesterol accumulates with both normal aging [36] and accelerated aging [34]. Other authors investigated the relationship between aging and cholesterol metabolism in the liver of 6-, 12-, 18- and 24-month-old male Wistar rats. They showed that the cholesterol concentration in the liver was not affected by aging, however, concentrations of the cholesterol precursors lanosterol and lathosterol increased, although desmosterol did not change [43].

## Cholesterol metabolism in the nervous system

The brain is the richest organ in cholesterol with 25% of the total body cholesterol. Cholesterol is synthesized in the central nervous system, as the blood-brain barrier prevents its entry into the brain in the form of lipoproteins [44, 45]. Cholesterol is present in exceptionally high amounts in the myelin sheaths surrounding neuronal axons representing 25% of the lipid molecules in the plasma membrane of brain cells, while various phospholipids, sphingomyelin and glycolipids make up the remainder. In addition to being a critical structural component for plasma membranes, cholesterol is able to regulate membrane trafficking and signal transduction pathways that start in the plasma membrane by stimulating or dimerizing receptors [6].

Importantly, disorders of cholesterol homeostasis in the adult brain are associated with different neurological diseases, such as Niemann-Pick [46], Alzheimer [47], Parkinson [48], Huntington [49] and epilepsy [50].

Since cholesterol-loaded lipoproteins cannot cross the blood-brain barrier, the brain has developed regulation of cholesterol based on synthesis and a specific catabolism by the enzyme CYP46A1. Cholesterol is cleared from the brain as 24S-hydroxycholesterol (24S-HC). The enzyme responsible for this oxidation is CYP46A1, located in cytochrome P450, and expressed in certain types of neurons, namely, pyramidal neurons of the hippocampus and cortex, Purkinje cells of the cerebellum, and hippocampal and cerebellar interneurons [51]. Due to the decrease in hydrophobicity, 24S-HC besides diffusing from the blood-brain barrier to be eliminated by the liver, is a potent bioactive molecule capable of modifying different cell functions. 24S-HC affects the cell survival rate [52–54], n-methyl D-Aspartate (NMDA) receptor activity [55, 56], exocytosis of vesicles [57] and LXR-induced transcriptional activity [58]. In addition to being the main by-product of brain cholesterol catabolism, 24S-HC is a potent bioactive molecule with therapeutic implications as well as a biomarker in different neurological disorders [59].

It is widely accepted that in the adult brain, cholesterol synthesis in neurons is extremely low. Therefore, to satisfy their physiological needs, neurons need to import cholesterol from astrocytes. These cells have the molecular mechanism to secrete cholesterol-enriched apolipoproteins, and neurons express surface receptors for families of proteins related to low-density lipoprotein (LRP) and low-density lipoprotein (LDL) receptors, which bind charged apolipoproteins and cholesterol [59].

## Cholesterol metabolism in the central nervous system in aging

As the brain ages, cognitive and motor performance decreases due to the accumulation of oxidative metabolism products. However, the aging brain contains few dead neurons, suggesting that aging must be accompanied by the activation or increase of neuronal survival mechanisms. Recent evidence points to the contribution of changes in membrane lipid composition to both age-dependent cognitive decline and robust neuronal survival [60].

Studies carried out in the human and rodent brain suggest that aging affects the cholesterol content in different regions, with the magnitude of variations depending on the analyzed sample, that is, total fraction, membrane fraction, lipid raft or synaptic fraction [6, 61, 62].

A moderate loss of brain cholesterol, both *in vitro* and *in vivo*, occurs in the hippocampal neuron membranes of elderly rodents [6, 63]. These findings agree with others reporting reduction in cholesterol levels from the age of 20 years onwards in the frontal and temporal cortices [64], and with a slight but significant reduction in the human hippocampus and cerebellum [62] or in brain synaptosomes derived from aged mice [6, 65]. Consistently, diminished cholesterol synthesis has been detected in the age-dependent human hippocampus [66] and increased levels of cholesterol 24-hydroxylase, the enzyme responsible for removing cholesterol in the brain [67] have been observed in the human brain and aged mice and in hippocampal neurons aging *in vitro* [63]. Furthermore, elevated levels of 24-HC were found in the plasma of elderly individuals [68].

Together, these results suggest that aging is accompanied by decreased synthesis and increased catabolism of cholesterol. However, changes in the amount of this lipid are highly variable during aging, ranging from no change to a 40% reduction [62].

The fluidity and asymmetry of cholesterol in the synaptic plasma membranes are altered in the aged mice with relative enrichment of cholesterol in the exofacial leaflet of synaptic membranes compared to young mice [69]. Increased cholesterol concentrations have been described in whole brain extracts from aged rats [70]. Together, these findings suggest that cholesterol levels respond differently during aging, depending on the metabolic needs of specific brain areas.

Relationship between aging and cholesterol metabolism in cortex and hippocampus, as well as in serum and liver of 6, 12, 18 and 24-month-old male Wistar rats showed that during aging the concentrations of the cholesterol precursors lanosterol, lathosterol and desmosterol do not change in the cortex, except for desmosterol which decreased 44% in 18-month-old rats. In the hippocampus, aging was associated with a significant reduction in lanosterol and lathosterol concentrations at 24 months (28 and 25%, respectively), as well as a significant decrease in desmosterol at 18 and 24 months (36 and 51%, respectively) [43].

Although some studies have proposed an association between hypercholesterolemia and sporadic Alzheimer’s Disease, the assumption that high concentrations of peripheral cholesterol impair the brain function remains controversial. In fact, the estimated number of adults in the US with cholesterol concentrations of 200 mg/dL or more is 99 million, and of that 32 million people have cholesterol levels of 240 mg/dL or more [71] whereas it is estimated only 5 million have Alzheimer’s Disease [72]. Thus, if high serum cholesterol concentrations were a risk factor for Alzheimer’s Disease, the incidence and prevalence of Alzheimer’s Disease should be much higher. However, recent studies suggest that there may be a non-linear relationship between plasma cholesterol levels and cognitive functions [73–76].

The most abundant apolipoproteins in the brain are ApoE and ApoA1, while the liver produces and secretes a large set of apolipoproteins with specific functions. A study using human stem cell-derived astrocytes and neurons highlights the importance of apoE in transporting cholesterol from astrocytes to neurons in the etiology of sporadic Alzheimer’s Disease, an aging-related dementia [77]. ApoE KO mice show synaptic loss, cognitive dysfunction, and high plasma lipid levels that can affect the brain function. In ApoE KO mice, plasma concentrations of cholesterol and phytosterols (campesterol and sitosterol) are high. Cholesterol precursors (desmosterol and lathosterol) not detected in the plasma of control mice, are measurable in ApoE KO mice. Amounts of brain cholesterol, desmosterol, campesterol and 24S-HC are significantly lower in ApoE KO mice compared to wild-type controls. These results demonstrate that brain cholesterol content, rate of synthesis (desmosterol) and 24S-HC export are reduced in ApoE KO hypercholesterolemic mice [78].

CYP46A1 KO mice develop a considerable reduction in the concentration of 24S-HC in the brain, without another cholesterol metabolite to compensate for this oxysterol. In these mice, cholesterol concentrations are similar to those in wild-type animals. However, the concentrations of desmosterol, the precursor of cholesterol, and its metabolite formed by the closure of the mevalonate, 24S, 25-epoxycholesterol pathway are reduced [79].

In a study aiming to elucidate the etiology of sporadic Alzheimer’s Disease, cholesterol and oxysterol concentrations were measured in the brain of ApoE ε2, ε3 and ε4 humans and knock-in mice at 8 weeks and 1 year of age. No effect of ApoE genotype or age on cerebral cholesterol or on 24S-HC has been demonstrated. Concentrations of 27-HC were elevated in 1-year-old animals for all ApoE genotypes [80]. Interestingly, lathosterol, a marker of cholesterol synthesis, was significantly reduced in 1-year-old animals for all ApoE genotypes. In addition, Apoε4 expressing mice exhibited lower concentrations of lathosterol compared to Apoε2 at both ages, and oxidized cholesterol metabolites were lower in Apoε2 mice compared to other genotypes at 8 weeks of age [80].

## Cholesterol metabolism is simultaneously altered in the liver and nervous system in aging

Aging induces changes in cholesterol metabolism in the brain and liver of rats. In an experimental model, there was an age-induced increase in cholesterol synthesis in the liver, demonstrated by an increase in the concentrations of lanosterol and lathosterol, and no change in the concentration of desmosterol. The amounts of these liver sterols were smaller than in the brain regions. In the cortex and hippocampus, desmosterol was the predominant precursor of cholesterol while in the liver, lathosterol was the most abundant precursor. This proportion remained stable during aging. This study showed that aging diminishes cholesterol synthesis in the hippocampus, demonstrated by a reduction in the concentration of desmosterol, which could reflect a reduction in age-related synaptic plasticity. The results showed that aging influences cholesterol synthesis in different ways in the brain and periphery, proving that cholesterol metabolism in the brain is autonomous [43].

Liver diseases, even in pre-cirrhotic stages, are also related to brain aging [81–87]. NAFLD was associated with poor cognitive performance independent of CVD and its risk factors when analyzing data from the Third National Health and Nutrition Examination Survey (NHANES III), covering a representative sample of the general US population investigating the relationships between NAFLD determined by ultrasound and cognitive impairment assessed by three computerized tests [84]. Decreased brain activity measured using infrared spectroscopy was observed in 24 female NAFLD patients compared to 15 healthy control females [85] and healthy middle-aged adults (67±9 years) in the descending cohort study of Framingham Heart Study NAFLD associated with lower total brain volume, independent of visceral adipose tissue and cardiometabolic risk factors, pointing to a possible link between hepatic steatosis and cerebral aging [86] Another study that evaluated the cross-sectional relationship of NAFLD and liver fibrosis with cognitive performance among Framingham Study participants aged 61±12 years, free from dementia and stroke and without excessive alcohol intake, showed that participants with high risk of advanced fibrosis had worse cognitive function compared with those at low risk [87].

NAFLD, characterized by accumulation of extra fat in liver cells that can lead to inflammation, liver fibrosis, cirrhosis, and liver cancer, is an obesity-related condition that has reached epidemic proportions [88, 89], and coexists with classic CVD risk factors [90]. A study in participants CARDIA study (Coronary Artery Risk Development in Young Adults) [91] that evaluated cognitive function and computed chest and abdomen tomography scans as part of the 25-year follow-up examination, and with cognitive function reassessed in the 30-year follow-up examination, showed that the inverse associations between NAFLD and cognitive scores were attenuated after adjustment for CVD risk factors, with the last predictor of worse cognitive performance both at baseline and throughout follow-up [92].

Ethical and methodological limitations do not allow direct measurements of synthesis and cholesterol in the brain and liver in humans. However, in rats, aging simultaneously induces changes in cholesterol metabolism in the brain and liver [43]. While the results showed an increase in cholesterol synthesis in the liver, in the brain there was a decrease in the hippocampus of aged rats. This study corroborates those where NAFLD was associated with brain aging characterized by cognitive impairment, reduced brain activity, and decreased brain volume [81–87], confirming that aging influences cholesterol synthesis in different ways in the brain and periphery, as well as that cholesterol metabolism in the brain is autonomous.

Alterations in the regulation of hepatic bile acid metabolism in metabolic syndrome [93, 94] and in non-alcoholic hepatic steatosis [95] have been reported in several investigations in humans, including in experimental models [96–98]. More recently, Alzheimer’s disease late onset has been suggested to be associated with metabolic syndrome [99]. In light of these results, it is not surprising that changes in cholesterol metabolism occur simultaneously with hepatic and neurological aging. Furthermore, in rats with aging, cholesterol accumulates in the liver, while the opposite occurs in the brain [43].

Neurological and hepatic alterations in cholesterol metabolism have been reported, the consequences of which for neurological diseases need to be explored. For example, bile acid metabolism appears to be decreased in dementia [100], and is related to Alzheimer’s disease [101]. Also, enzymes related to cholesterol metabolism, such as PCSK9, could be linked to Alzheimer’s disease [102]. Consequently, the connection of disorders of cholesterol metabolism in the liver and the central nervous system deserves investigation.

Future research should look for blood markers that identify the simultaneity of liver and neuronal aging.
